# Synthesis and Application of a Novel Multi-Branched Block Polyether Low-Temperature Demulsifier

**DOI:** 10.3390/molecules28248109

**Published:** 2023-12-15

**Authors:** Shaohui Jiang, Qingsong Li, Botao Xu, Tao Zou, Yan Zhang, Wei Ping, Qiang Ma

**Affiliations:** 1 State Key Laboratory of Heavy Oil Processing, China University of Petroleum East China, Qingdao 266580, China; b16030104@s.upc.edu.cn; 2 China Oilfield Services Limited, Tianjin 300450, China; xubt@cosl.com.cn; 3 Huabei Oilfield Company, China National Petroleum Corporation, Renqiu 062552, China; cy3_zout@petrochina.com.cn; 4 Drilling & Production Engineering Technology Research Institute, CNPC Chuanqing Drilling Engineering Company Limited, Xi’an 710018, China; zhangy_zcy@cnpc.com.cn; 5 Fujian Provincial Company of National Petroleum and Natural Gas Pipeline Network Group, Fuzhou 350000, China; pingwei@pipechina.com.cn; 6 CNPC Chuanqing Drilling Engineering Company Limited, Chengdu 610051, China; maqsx_sc@cnpc.com.cn

**Keywords:** block polyether demulsifier, thick oil, low-temperature demulsification, field experiment

## Abstract

In this paper, a low-temperature thick oil demulsifier with high polarity was prepared by introducing ethylene oxide, propylene oxide block, and butylene oxide using m-diphenol as a starting agent. The main reasons for the difficulty involved in the low-temperature emulsification of extractive fluids were explained by analyzing the synthetic influencing factors and infrared spectra of the star comb polymer (PR-D2) and by analyzing the four fractions, interfacial energies, and zeta potentials of crude oils from the Chun and Gao fields. The effects of PR-D2 surfactant on the emulsification performance of crude oil recovery fluids were investigated via indoor and field experiments. The experimental results indicate that the optimal synthesis conditions for this emulsion breaker are as follows: a quality ratio of ionic reaction intermediates and meso-diphenol of R = 10:1; 1 g of the initiator; a polymerization temperature of 80 °C; and a reaction time of 8 h. Colloidal asphaltenes in the crude oil were the main factor hindering the low-temperature demulsification of the Gao oilfield’s extractive fluids, and the reason for the demulsification difficulty of the extractive fluids in the Chun oilfield is that the temperature of demulsification is lower than the wax precipitation point. The demulsification rate of the Chun oilfield’s extractive fluids reached more than 98% when the PR-D2 concentration reached 150 mg/L at 43 °C. The demulsification rate of the Gao oilfield’s extractive fluids reached more than 98% at a PR-D2 concentration of 150 mg/L at 65 °C. The field experiments show that the Chun oilfield’s extractive fluids can still demulsify after the temperature is reduced to 43 °C in winter. The emulsification temperature of the Gao oilfield’s extractive fluids was reduced from 73 °C to 68 °C, with an excellent demulsification effect.

## 1. Introduction

Crude oil demulsifiers are an essential chemical reagent for crude oil dewatering treatment in oilfields and refineries [1,2]. With major oilfields reaching a high-water-content production period, the water content of crude oil is increasing each year [3,4]. Three times oil extraction technology, heavy oil extraction technology, and various measures to increase production are widely utilized, resulting in the more complex nature of the extracted fluid, the stronger stability of the crude oil emulsion, and difficulties in demulsifying and dewatering [5]. Compared to thin oil, thick oil with a higher viscosity is more difficult to dewater via chemical methods, and conventional demulsifiers have a poor effect on the low-temperature dewatering of thick oil, resulting in a higher dewatering temperature each year [6]. The heating energy consumption in the demulsification and dewatering process is sustainably rising, and costs have increased significantly [7].

The essence of demulsification is the process of the droplets in the emulsion aggregating with each other under the action of a demulsifier [8]. The resistance of an emulsion to demulsification mainly consists of electrostatic repulsion force, demulsifier diffusion resistance, and interfacial film strength, in which the surface charge of the droplet does not correlate well with temperature [9,10]. After delivery to the treatment station, the viscosity of the extracted fluid increases significantly, reducing the ability of the demulsifier to diffuse [8,11,12]. Meanwhile, colloidal asphaltenes, waxes, and admixtures at low temperatures increase the interfacial film strength in the extracted fluid, enhancing the difficulty of demulsification [13,14,15,16]. Therefore, the development of a low-temperature demulsifier should focus on reducing the diffusion resistance of the demulsifier and the interfacial film strength under low-temperature conditions [17,18].

Butylene oxide and propylene oxide (EO/PO) block polyether is a typical nonionic polymer surfactant with a rich structural designability, which can be assembled into meso-structures with various morphologies in solvents [19,20,21]. Presently, there are many types of commercialized block polyethers, including linear structures [22], tetronic and tetronic R [23], dendritic polyethers [24], star polyethers [25], tree polyethers [20], and other non-linear block polyethers [26], among which the multi-dendritic block polyethers with more active end-groups have excellent performance in reducing the interfacial tension of water [20]. The type of initiator, the degree of branching, the type and order of blocks, and the mass ratio of EO/PO all affect the demulsifying properties of dendritic polyether surfactants [27,28]. The type of initiator affects the degree of branching of the surfactant, which further influences the concentration of active end groups [29]. Al-Sabagh synthesized a kind of ethyl-polyacryloxycoolaldehyde resin demulsifier and investigated the factors influencing the efficiency of the demulsifier [30]. The demulsifying properties of demulsifiers increase with increasing concentration of the demulsifier, alkyl chain length, and water content, but asphaltenes limit the demulsification efficiency [18,31]. The demulsifying performance of tree-type block polyethers increased with the decrease in PEO content, and the ratio of the initiator and PPO had a slight effect on their demulsifying effectiveness [24]. The higher the molecular weight of the emulsion breaker, the stronger the effect of demulsifying and dewatering, and for the same molecular weight of the demulsifier, the number of branched chains does not impact its demulsifying effect [32]. With the increase in the interfacial stability of the extracted fluid, the difficulty of demulsification in low-temperature environments increases dramatically [33]. Although the research on novel thick-oil demulsification has broad application prospects, there is still a need for further research on highly efficient low-temperature demulsifiers with a more complex degree of branched chains and a higher molecular weight.

In this paper, a star comb polymer was prepared using m-diphenol as a starting agent and EO/PO block, and then the star comb polymer (PR-D2) was prepared by introducing epoxy butane modification, and its structure was characterized by infrared spectroscopy. The emulsification performance of PR-D2 on the extractive fluids of the Chun and Gao oilfields was investigated by means of indoor and field experiments, and the reasons for the low-temperature difficulty of emulsification were evaluated by analyzing the four components of crude oil, interfacial properties, and zeta potential, and the reasons why PR-D2 could realize low-temperature emulsification were explained.

## 2. Results and Discussion

### 2.1. Synthesis and Characterization of PR-D2

**Figure 1:**
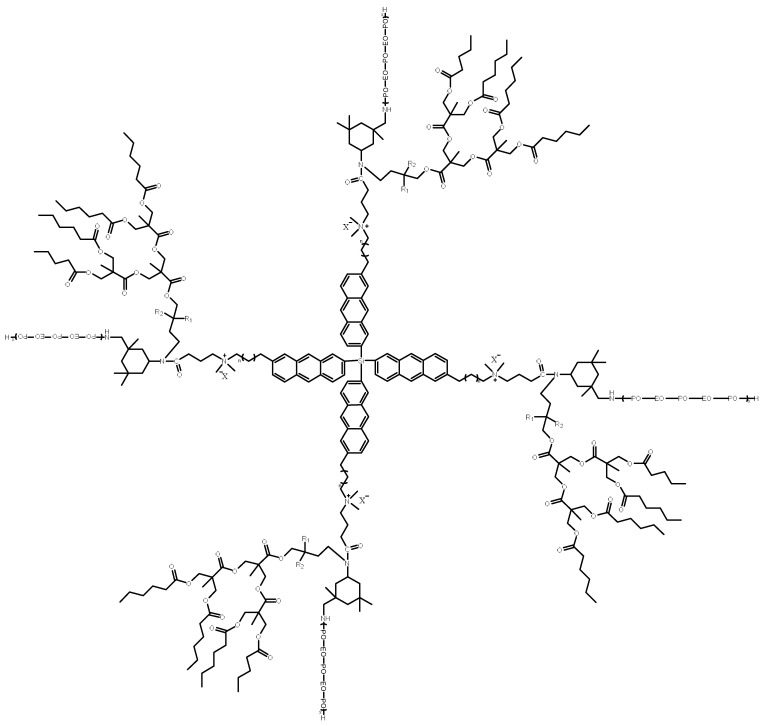
The chemical molecular formula of the star comb surfactant (PR-D2).

**Figure 2:**
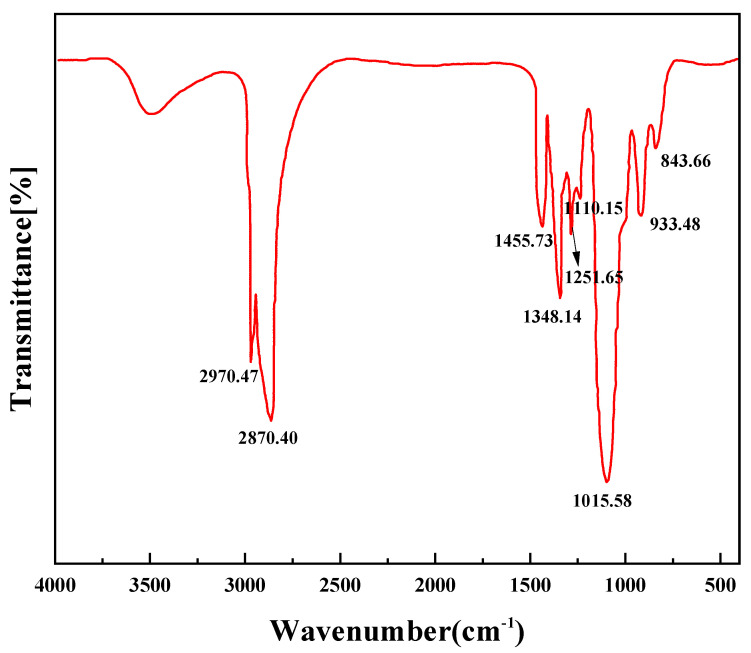
PR-D2’s infrared characterization.

Figure 1 and Figure 2 show the molecular formula and IR characterization of the PR-D2 surfactant, respectively. Figure 1 shows the molecular formula of the PR-D2 demulsifier the main chain is constructed with resorcinol, the branched chain is constructed with EO/PO, and the branched chain has electronegative ions, which endows the demulsifier with high permeability performance in thick oil. As shown in Figure 2, the stretching vibration of C-O-C caused an absorption peak at 1110.15 cm^−1^, and the stretching vibration of -CH_3_ caused a strong absorption peak at 2970.47 cm^−1^, which preliminary serves as evidence for the fact that EO and PO react with the -OH of the propylene oxide molecule. The absorption peak at 2870.40 cm^−1^ is generated by the stretching vibration of R-CH_2_-NR_2_, and the absorption peak at 1455.73 cm^−1^ is caused by the bending vibration of -CH_2_-, which tentatively indicates that -CH_2_ is connected to N. The absorption peak is caused by the C-N stretching vibration at 1348.14 cm^−1^, and the out-of-plane bending of C-H on the benzene ring at 843.66 cm^−1^ further indicates that the C-N bond is attached to the benzene ring. The C-O-C stretching vibration of the aryl ether at 1251.65 cm^−1^, the C-O-C stretching vibration at 1110.15 cm^−1^, the -OR stretching vibration of the aryl ether at 1015.58 cm^−1^, and the C-O stretching vibration of the epoxy compound at 933.48 cm^−1^ further indicate that the epoxy ether and epoxy propyl ether are formed.

### 2.2. Analysis of Factors Affecting Emulsion Breakage in Oilfield Samples

With the deepening of oilfield development, the variety of chemical used for demulsification has gradually increased. A combination of different factors enhances the stability of the emulsion, making the type of extracted crude oil emulsion more complex and increasing the difficulty of crude oil dewatering treatment. Therefore, by studying the nature of the extracted liquid system, the emulsion-breaking mechanism is explored in a targeted way.

#### 2.2.1. Analysis of Emulsification Properties of Crude Oil Components

**Figure 3:**
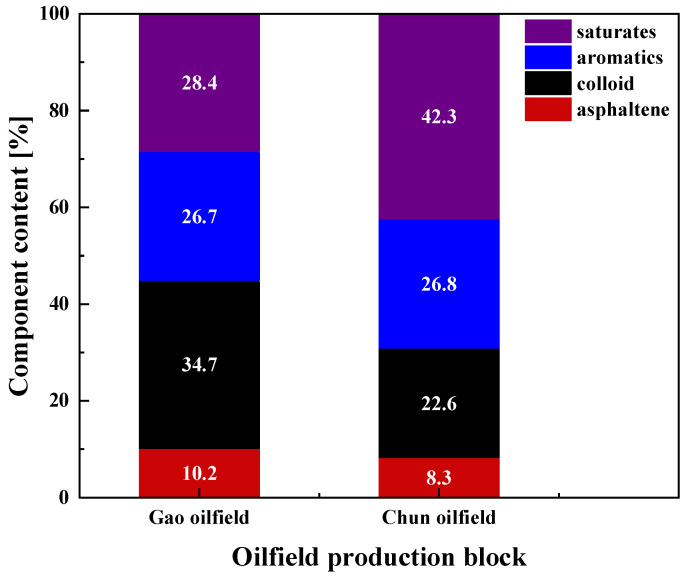
Analysis of the four components of the crude oil.

Figure 3 shows the four components of the crude oil of the Gao oilfield and the Chun oilfield. The crude oil’s emulsion stability depends largely on the nature of the interfacial film formed by the adsorption of natural emulsifiers, such as asphaltenes, on the oil–water interface. The crude oil gum/asphaltene of the Gao oilfield is higher than 3:1, which is conducive to the formation of an emulsion in the crude oil recovery fluid, and thus emulsion dewatering is difficult. The gum and asphaltene content of Chun oilfield crude oil is more than 30%, which makes dewatering more difficult at low temperatures.

#### 2.2.2. Study of Interfacial Properties of Crude Oil Components

**Figure 4:**
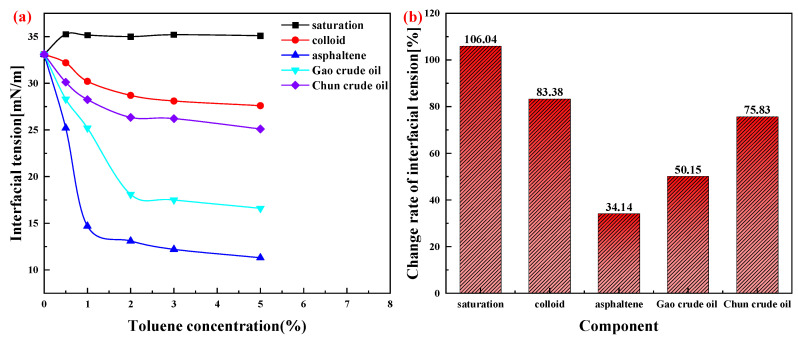
The relationship between interfacial tension and the concentration of toluene in the crude oil of Gao oilfield and Chun oilfield. (**a**) Effect of toluene content on the surface tension of components and crude oils. (**b**) Rate of change in interfacial tension at 5 wt% toluene.

Figure 4 shows the interfacial tension curves of crude oil, colloids, asphaltenes, saturated fractions, and water after dilution with different concentrations of toluene solution. In the conventional components, colloid and asphaltene contain more heteroatoms and polar groups, thus having higher polarity and interfacial activity. Compared to colloids, asphaltenes have higher polarity, and the interfacial tension between the asphaltene–toluene solution and water is lower. The saturated fraction of crude oil contains waxes, which are high in straight-chain alkanes and isoparaffins, and their interfacial activity is poorer than that of polar groups, resulting in higher interfacial tensions than those of gums and asphaltenes. The interfacial tension of the crude oil–toluene mixture is smaller compared to the colloid–toluene mixture.

The surface tension of the colloid-saturated fraction–toluene mixed solution should be higher than the colloid–toluene mixed solution, but the interfacial tension of the crude oil formed by mixing with asphaltene was significantly lower than that of the crude oil created via colloid and saturated fraction mixing, indicating that asphaltene is the main adsorption-active component of the interface and the major active substance for the stabilization of the extracted fluid. Figure 4 shows that the interfacial tension decreases as the asphaltene and colloid contents increase, indicating that the high content of colloidal asphaltenes is the main reason for the difficulty involved in low-temperature emulsion breaking. Figure 3 shows that the crude oil of the Gao oilfield has a higher content of colloidal asphaltenes, and its emulsification is more difficult. Figure 4b shows the rate of change in interfacial tension of each component mixed with 5 wt% toluene, and the surface tension of asphaltene/toluene decreases to 34.14%, which also proves that the higher the content of colloidal asphaltene, the more difficult it is to break the emulsion.

#### 2.2.3. Analysis of Wax Content of Crude Oil

Table 1 shows that the wax content in the crude oil of the Gao oilfield and the Chun oilfield is higher than 10 wt%, representing high-wax-content crude oil. The higher the wax content in crude oil, the easier it is for waxes to be crystallized at low temperatures, and the wax crystals with a high concentration will easily interconnect to form a solid space network structure, which will increase the freezing point of the crude oil and reduce its fluidity, and therefore the wax must be considered when breaking the emulsion at a low temperature. The temperature range of the wax precipitation point of high-wax-content crude oil is 40~50 °C. The influence of wax can be disregarded in the Gao oilfield because the dewatering temperature at the high green site exceeds 60 °C. Meanwhile, the lowest dewatering temperature of the Chun oilfield crude oil is 43 °C, and the wax in the crude oil will crystallize out, indicating the breaking effect of the crude oil.

#### 2.2.4. Charge Potential Analysis of Emulsions

The zeta potentials of the extractive fluid were determined using a potential analyzer. Table 1 shows that the surface of the emulsion of the Gao oilfield’s and the Chun oilfield’s extracted liquid is negatively charged. The larger the absolute value of the zeta potential, the larger the thickness of the diffusion layer, the greater the stability of the emulsion, and the more difficult the emulsion treatment is to break. Therefore, from the results of the zeta potential test, the emulsion of the Gao oilfield’s extraction fluid is more difficult to break.

### 2.3. PR-D2 Dehydration Performance of Different Reaction Conditions

**Figure 5:**
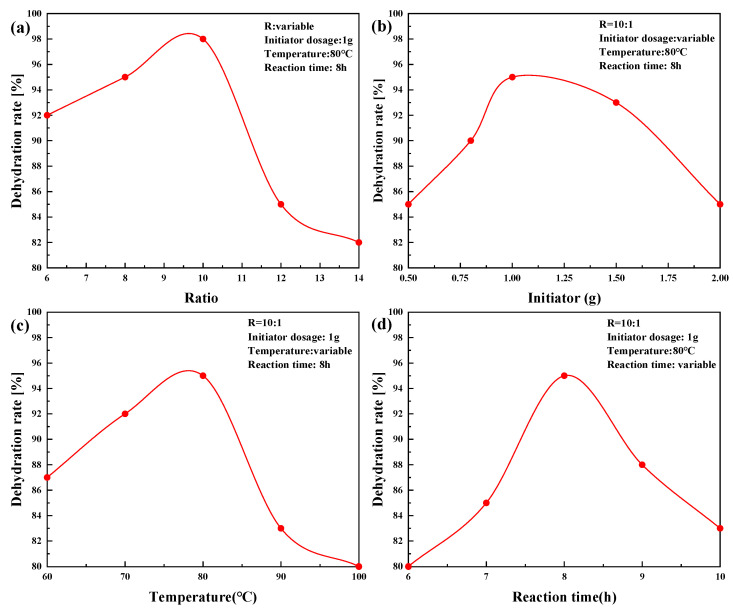
Influence of synthetic factors on the demulsifying properties. (**a**) Effect of the mass ratio of ionic reaction intermediates and meso-diphenol on the performance of the demulsifier. (**b**) Effect of the initiator dosage on the performance of the demulsifier. (**c**) Effect of the polymerization temperature on the performance of the demulsifier. (**d**) Effect of the reaction time on the performance of the demulsifier.

Figure 5 shows the influence of R (R is the mass ratio of ion intermediates and resorcinol), initiator dosage, synthesis temperature, and reaction time on the dehydration properties of the PR-D2 surfactant, respectively. The emulsion breaking temperature was set at 45 °C, the dosage of PR-D2 was 200 mg/L, and the emulsion breaking time was 3 h.

Figure 5a shows the effect of PR-D2 on the dewatering rate of the Chun oilfield’s crude oil for R = 6, 8, 10, 12, and 12, and the optimum value of the effect of the emulsion breaker is reached when R = 10. Figure 5b shows the effect of initiator dosage on the dehydration effect of PR-D2. With the increase in the initiator dosage, the dehydration of the product first increases and then decreases. Attributed to the increase in the initiator dosage, the density of free radicals in the system increases, resulting in a higher polymerization rate and an increase in product conversion. The dosage of the initiator has a great influence on the molecular weight of the product—the greater the initiator dosage, the larger the molecular weight of the product. Meanwhile, the molecular weight has a greater effect on the product of crude oil emulsification and dehydration, so the optimal dosage of the initiator is 1 g. Figure 5c shows that with an increasing polymerization reaction temperature, the crude oil dehydration rate of the synthesized product first increases and then decreases, attributed to the decomposition activation energy of the initiator drastically increasing the total activation energy of the synthesis reaction. When increasing the temperature of the synthesis reaction, the number of activated molecules of the initiator increases dramatically, thus increasing the density of free radicals in the system and accelerating the polymerization reaction. However, when the synthesis reaction temperature is excessively heart, the free radical density is excessively high and the product’s molecular weight is reduced, which will decrease the emulsification and dewatering rate of the surfactant on crude oil. Figure 5d investigates the effect of the reaction time on the demulsifying properties of the surface activator. When the reaction time increases, the conversion and the molecular weight of the synthesized product increases, and therefore the crude oil dehydration rate increases. After the reaction time exceeds 8 h, due to the excessive molecular weight of the synthesized product, its diffusion difficulty increases due to the crude oil’s diffusion to the oil–water interface, and the dehydration rate decreases. In summary, the suitable conditions for PR-D2 synthesis are as follows: R = 10; 1 g of the initiator; a polymerization temperature of 80 °C; and a reaction time of 8 h.

### 2.4. PR-D2 Demulsification Performance

The low-temperature demulsification performance of the PR-D2 surfactant can be reflected by the rate of crude oil dehydration and the cleanliness of the aqueous phase after demulsification. In this paper, the demulsification and dewatering performance of PR-D2 surfactant on crude oil emulsions from the Chun oilfield and Gao oilfield was investigated under different temperature conditions.

#### 2.4.1. Demulsification and Dehydration Performance of PR-D2 on Chun Oilfield Crude Oil

**Figure 6:**
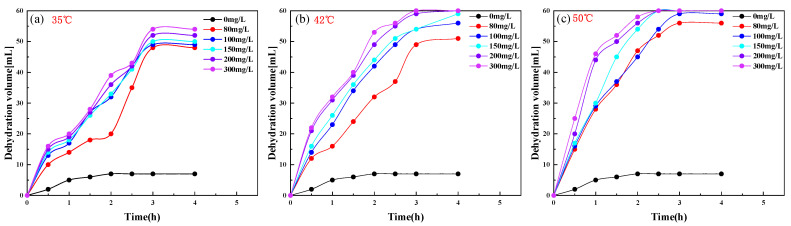
Influence of the PR-D2 surfactant on the crude oil of the Chun oilfield.

Figure 6a–c show the effect of the PR-D2 surfactant on the emulsion breaking effect of Chun oilfield crude oil at different concentrations at 35 °C, 43 °C, and 50 °C, respectively, and the water content (*W_v_*) of Chun oilfield crude oil was 60%, while the crude oil emulsion volume (*V*_0_) was 100 mL. Figure 6 shows the enhancement of the breaking and dewatering properties with increasing PR-D2 concentration and increasing breaking temperature. Table 2 shows that without the addition of a demulsifier, the rate of dewatering (*S*) was 11.6%, and the cleanliness of the dewatered water was ranked as class II at a demulsification temperature of 35 °C. Figure 6a shows that S is 90% when the dosage of the emulsion breaker is 300 mg/L, and the water is not completely removed. The reason for this is as follows. The diffusion speed of PR-D2 in crude oil is low at low temperatures. In addition, since the emulsification temperature is lower than the wax precipitation point, the wax precipitation is retained in the water droplets, separating the water droplets and hindering the agglomeration of the aqueous phase, which increases the difficulty of emulsification. Our results show that at 35 °C, the PR-D2 emulsion breaker has a weak emulsion breaking effect on the crude oil emulsion of the Chun oilfield. When the breaking temperature increased to 42 °C and 50 °C, the dosage of PR-D2 was 200 mg/L and 150 mg/L, respectively, which could realize the complete dewatering of the crude oil emulsion, while the cleanliness of the dewatered aqueous phase was ranked as class Ⅰ. Under a temperature of 43 °C, the dewatering rate of PR-D2 can reach more than 98% when the concentration of PR-D2 exceeds 150 mg/L, which can fulfill the requirements of the execution experiment.

#### 2.4.2. Demulsification and Dehydration Performance of PR-D2 on Gao Oilfield Crude Oil

**Figure 7:**
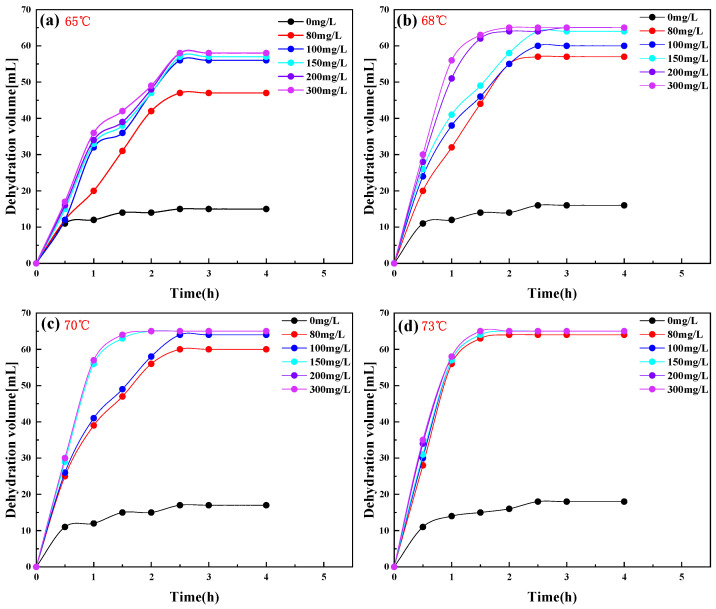
Influence of the PR-D2 surfactant on the crude oil of the Gao oilfield.

Figure 7a–d show the influence of the PR-D2 surfactant on the emulsion breaking effect of Gao oilfield crude oil at different concentrations at 65 °C, 68 °C, 70 °C, and 73 °C, respectively, and the water content (*W_v_*) of Chun oilfield crude oil was 65%, while the crude oil emulsion volume (*V*_0_) is 100 mL. Figure 7 shows the excellent demulsification and dewatering effect of crude oil with an increasing PR-D2 concentration and temperature. The temperature has a stronger influence on the effect of emulsification—the higher the emulsification temperature, the more rapid the emulsification speed, and the lower the concentration of PR-D2. Table 3 shows that the rate of dewatering (*S*) was 21.3%, 24.6%, 26.2%, and 27.7%, respectively, without the addition of PR-D2, and the cleanliness of the dewatered water under a temperature environment of 65 to 73 °C was ranked as class I. When the temperature reaches 73 °C and the dosage of the demulsifier is 80 mg/L, the rate of demulsification and dehydration is more than 98%. The dewatering rate of the Gao oilfield’s crude oil is more than 98% when the dewatering temperature is more than 68 °C and the dosage of the PR-D2 agent is 150 mg/L.

### 2.5. Field Experiment

**Figure 8:**
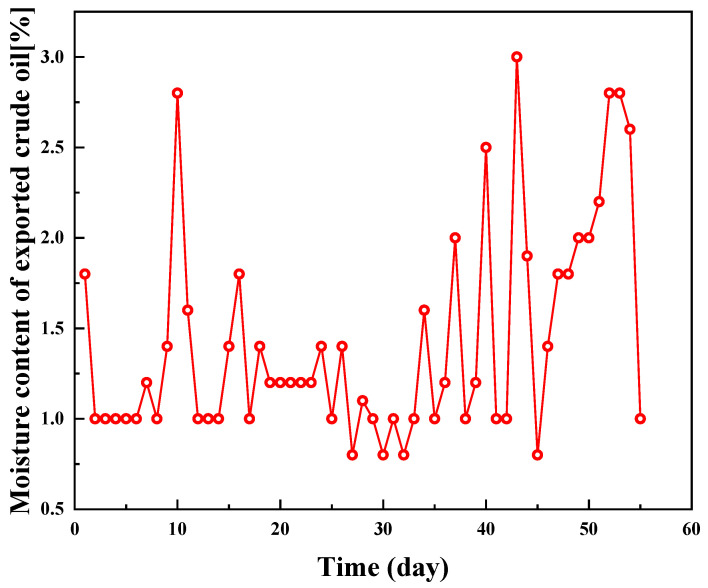
Water content curve of the exported crude oil after treatment at the Gao oilfield.

**Figure 9:**
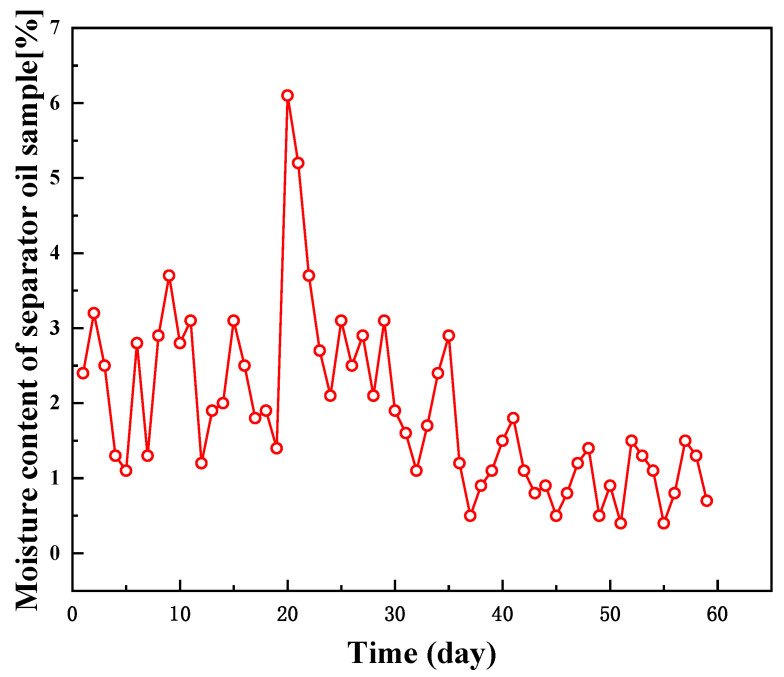
Water content curve of the exported crude oil after treatment at the Chun oilfield.

The current breaking and dewatering temperatures in the Gao and Chun oil fields are 78 °C and 50 °C, respectively, which are too high, and it is hoped that the processing temperatures of crude oil can be lowered to 68 °C and 35 °C, respectively, without lowering the dewatering rate. Figure 8 and Figure 9 show the water content curves of crude oil exported from the Gao oilfield and the Chun oilfield, respectively. The Gao oilfield’s treatment liquid volume was around 2000 m^3^/d, and the external oil transmission is around 220,000 kg/d. The PR-D2 concentration was 150 mg/L (300 kg/d) at 68 °C. In winter, the experimental temperature of the Gao oilfield dropped gradually, from 73 °C to 65 °C, and after the addition of PR-D2, the water content was always kept below 2.8%, indicating that the dewatering rate of the crude oil is excellent, and it can fulfill the Gao oilfield’s requirements. The experimental temperature in the Chun oil field gradually reduced from 50 °C to 35 °C. After the agent was injected on the 20th day, the water content in the exported crude decreased rapidly and remained below 3%, and the trend showed positive performance.

## 3. Materials and Methods

### 3.1. Materials

Analytical reagent-grade epoxy-butane, propylene oxide, alkyl benzene sulfonic acid, mono-ethanolamine, m-phenol, potassium hydroxide, and xylene were obtained from Aladdin Chemical Co., Ltd. (Shanghai, China). All of these chemicals were used as received, without any purification. The emulsified thick oil used in this study was obtained from the Chun and Gao oilfield blocks of the Shengli oilfield (Dongying, China) in China, and the compositions of these samples are shown in Figure 3.

### 3.2. Synthesis of Demulsifiers

The synthesis of the PR-D2 surfactant was mainly divided into two steps: (1) 1 wt% catalyst potassium hydroxide was placed into the reactor kettle, the air in the kettle was replaced with nitrogen, then the kettle was pumped to a negative pressure and stirring was commenced. After the kettle temperature rose from 20 °C to 120 °C, the kettle was again pumped to a negative pressure. The kettle temperature was controlled to be 115 °C to 125 °C, and epoxybutane and epoxypropane were continuously passed into the kettle proportionally, and the reaction was kept stable for 0.5 h. Then, we continuously passed through ethylene oxide, keeping the kettle pressure at 0.3 MPa or below. Then, when the pressure in the reaction kettle was reduced to 0 MPa, alkyl benzene sulfonic acid monoethanolamine was added at a ratio of 5:1, and the mixture was stirred well to obtain the emulsion-breaking agent ionic reaction intermediates. (2) The PR-D2 demulsifier was synthesized by adding meso-diphenol and ionic reaction intermediates, 1 g of the initiator, benzoyl peroxide, and 50 mL of the solvent, xylene, into a four-necked flask and then polymerizing this mixture at 80 °C for 8 h.

### 3.3. Characteristics of PR-D2

The structure of the synthetic emulsion breaker was characterized by WQF-520 FR (Beijing Rayleigh Analytical Instruments Co., Ltd., Beijing, China) with an infrared DTG detector in the range of 4000–400 cm^−1^ with 16 scans and a resolution of 4 cm^−1^.

### 3.4. Interfacial Tension

The surface tension of the samples was determined using a fully automated surface tension meter (BZY100/BZY200, Shanghai Fangrui Instrument Co., Shanghai, China). Samples were added to the cell and left for 5~10 min at 25 °C to bring the system to equilibrium for measurement, and each test was repeated at least 3 times to obtain the average value.

### 3.5. Four-Component Measurement and Crude Oil Wax Content Measurement

**Figure 10:**
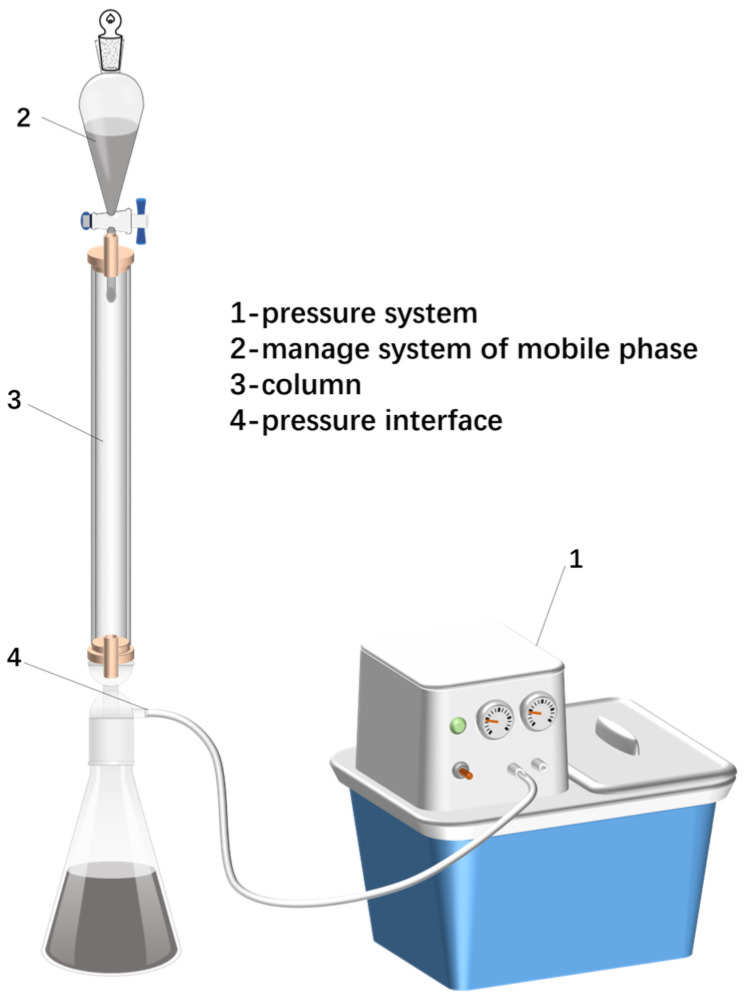
Schematic diagram of the column chromatography experimental setup.

Samples from the Gao oilfields and Chun oilfields with different recovery times were taken to measure the contents of the four components and wax. The asphaltene in crude oil is insoluble in n-hexane, while the other components of heavy oil can be dissolved in n-hexane. So, the asphaltene in the heavy oil was first cleaned out, and then the crude oil was passed through a silica gel alumina chromatography column (Figure 10), using different solvents to rinse out the gums, saturated hydrocarbons, and aromatic hydrocarbons, and then the sample was weighed after the solvent had evaporated. Oil and wax were obtained from the crude oil after the removal of polar substances by means of alumina column chromatography. The fraction was precipitated via crystallization at −20 °C using a benzene–acetone (the ratio of volume was 1:1) solution as the dewaxing solvent.

### 3.6. Zeta Electric Potential

Samples obtained from the Gao oilfield and the Chun oilfield at different times were injected into the holding tank of the zeta potential device and then placed into the device. The instrument was set to Zetapotential mode through the software at 30 °C, the equilibrium time was set to 2 min, and measurements were taken 3 times.

### 3.7. Demulsification Performance Measurable

The prepared crude oil emulsion was poured into a stoppered measuring cylinder at the 100 mL scale, and a thermostatic water bath was heated up to the corresponding experimental temperature. The stoppered measuring cylinder containing the crude oil emulsion was placed in a thermostatic water bath with the bath level above the crude oil emulsion level and preheated for 15 min to increase the temperature of the emulsion to the predetermined dehydration temperature. An amount of PR-D2 demulsifier was added to the cylinder using a pipette. After tightening the cap, we turned the stoppered cylinder upside down 3 to 5 times, slowly loosened the cap to deflate the cylinder, and then tightened the cap again, after which we manually oscillated the cylinder up and down vigorously (100 ± 10) times, with an amplitude of more than 10 cm. After mixing completely, we loosened the cap and placed the stoppered cylinder back in the thermostatic water bath to settle. The amount of effluent removed at different times was recorded and the dewatering rate was calculated using Equation (1).

(1) $$S=\frac{V}{V_{0}\times W_{V}}\times 100\%$$

where *S* is the crude oil demulsifier dehydration rate, in %; *V* is the crude oil emulsion effluent amount after settling, in mL; *W_V_* is the crude oil emulsion volumetric water content; and *V*_0_ is the crude oil emulsion volume, in mL.

The test temperature of the crude oil from the Chun oilfield is 35 °C, 42 °C, and 50 °C, respectively, and the test temperature of the crude oil from the Gao oilfield is 65 °C, 68 °C, 70 °C, and 73 °C, respectively. The design of the test temperature is based on the construction parameters of the oilfield.

## 4. Conclusions

In this paper, a new dendritic polymer PR-D2 surfactant was synthesized. The optimal synthesis conditions for this emulsion breaker are as follows: R = 10; 1 g of the initiator; a polymerization temperature of 80 °C; and a reaction time of 8 h. The properties of the Chun oilfield’s and the Gao oilfield’s extractive fluids were analyzed, and it was found that colloidal asphaltenes in the crude oil were the main factor hindering the low-temperature demulsification of the Gao oilfield’s extractive fluids. The reason for the demulsification difficulty in the Chun oilfield is that the temperature of demulsification of the extractive fluids is lower than the wax precipitation point. The experimental results indicate that the demulsification rate of the Chun oilfield’s extractive fluids could reach more than 98% when the PR-D2 concentration reached 150 mg/L at 43 °C. The demulsification rate of the Gao oilfield’s extractive fluids reaches more than 98% with a PR-D2 concentration of 150 mg/L at 65 °C. Field experiments show that the Chun oilfield can still demulsify the extractive fluids after the temperature is reduced to 43 °C in winter. The emulsification temperature of the Gao oilfield’s extractive fluids was reduced from 73 °C to 68 °C, with an excellent demulsification effect.

## Figures and Tables

**Table 1:** The emulsion sample’s zeta electric potential and the crude oil wax content of the Gao oilfield and the Chun oilfield.

Time (d)	Sample Source	Test Temperature (°C)	Zeta Electric Potential (mV)	Crude Oil Wax Content (%)
1	Gao oilfield	30	−17.3	13.2
	Chun oilfield	30	−8.6	15.6
27	Gao oilfield	30	−18.2	13.7
	Chun oilfield	30	−9.4	16.1
75	Gao oilfield	30	−17.5	13.5
	Chun oilfield	30	−9.1	15.5

**Table 2:** Dehydration rate of the Chun oilfield’s crude oil with different concentrations of PR-D2.

Code	Temperature (°C)	Dehydration Rate (%)						Cleanliness
		0 mg/L	80 mg/L	100 mg/L	150 mg/L	200 mg/L	300 mg/L	
a	35	11.6	80.0	81.6	83	86.7	90.0	Ⅱ
b	42	11.6	80.0	93.3	98.3	100.0	100.0	Ⅰ
c	50	11.6	93.3	98.3	100.0	100.0	100.0	Ⅰ

**Table 3:** Dehydration rate of the Gao oilfield’s crude oil with different concentrations of PR-D2.

Code	Temperature (°C)	Dehydration Rate (%)						Cleanliness
		0 mg/L	80 mg/L	100 mg/L	150 mg/L	200 mg/L	300 mg/L	
c	65	23.1	72.3	86.1	87.7	89.2	89.2	Ⅰ
b	68	24.6	87.7	92.3	98.5	100.0	100.0	Ⅰ
c	70	26.2	92.3	98.5	100.0	100.0	100.0	Ⅰ
d	73	27.7	98.5	100.0	100.0	100.0	100.0	Ⅰ

## Data Availability

No new data were created or analyzed in this study. Data sharing is not applicable to this article.
